# Reply to Ren et al.: The role of a liver-specific mitochondrial carrier SLC25A47 in glucose homeostasis

**DOI:** 10.1073/pnas.2307922120

**Published:** 2023-07-31

**Authors:** Jin-Seon Yook, Zachary H. Taxin, Satoshi Oikawa, Masanori Fujimoto, Shingo Kajimura

**Affiliations:** ^a^Department of Medicine, Division of Endocrinology, Diabetes, and Metabolism, Beth Israel Deaconess Medical Center and Harvard Medical School, Boston, MA 02115; ^b^Department of Endocrinology, Hematology, and Gerontology, Graduate School of Medicine, Chiba University, 260-8670 Chiba, Japan; ^c^Department of Molecular Diagnosis, Graduate School of Medicine, Chiba University, 260-8670 Chiba, Japan; ^d^HHMI, Chevy Chase, MD 20815

Here, we describe the following points to respond to Ren et al. (1). In short, the analysis by the authors has no impact on the conclusions of our work.

First, the absence of an expression quantitative trait loci (eQTL) signal for a target gene does not, in any way, imply absent importance. Since eQTLs and genome-wide association studies (GWAS) provide different information, they are more complementary than additive (2). In fact, only 2 to 8% of baseline expression of trait-related genes can explain GWAS associations (3). Of note, we used the Type 2 Diabetes Knowledge Portal to explore human phenotype associations, which includes multiple, large genetic studies of human subjects with Type 2 diabetes, cardiovascular disease, and liver disease (4). In contrast, the eQTL database used by Ren et al. (1) was built on human postmortem tissues from a relatively small sample size (n = 97) with nondiseased subjects in the normal range for the donor‘s ages (5). Thus, it is not meaningful to compare these two distinct data sources with different predictive power and genetic distributions.

**Fig. 1.**
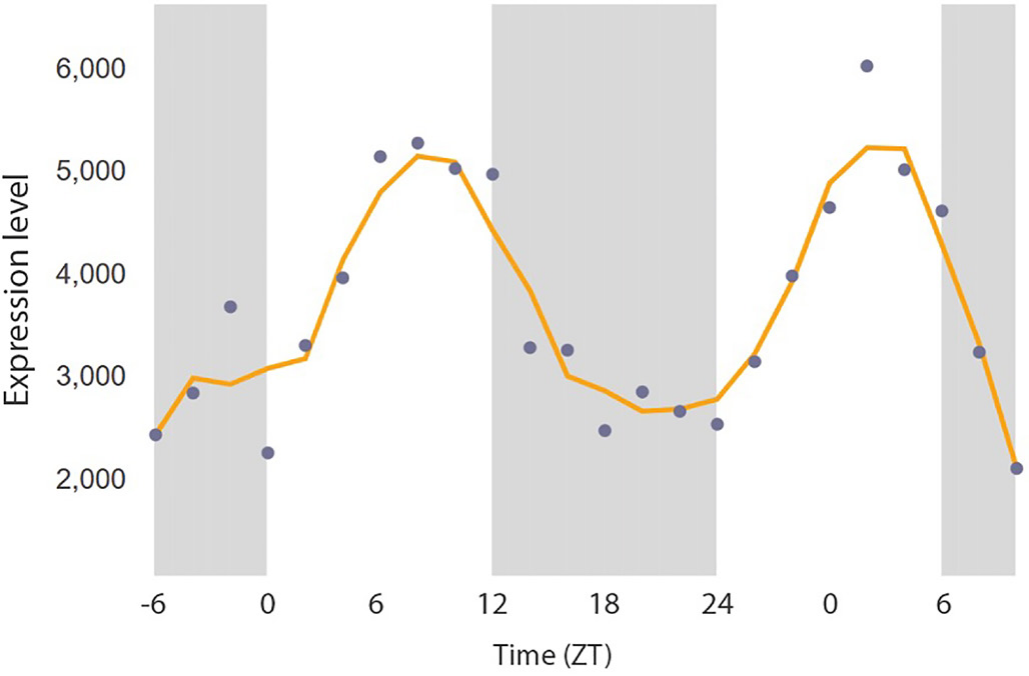
The expression of *Slc25a47* in mouse liver tissue throughout the day. The data are from CircaDB (http://circadb.hogeneschlab.org/mouse). The original data are deposited in RNAseq_NM_001012310.

Second, the expression of SLC25A47 is not static. For example, the circadian gene database CircaDB (6) found that *Slc25a47* exhibits a dynamic circadian expression pattern (>twofold change) in mouse liver (Fig. 1). Importantly, our functional studies demonstrated that a change in *Slc25a47* expression by 50% was enough to significantly alter hepatic glucose production in vivo (4). Accordingly, collection time and possibly nutritional status of the samples substantially influence *SLC25A47* expression levels; however, these data are missing from the eQTL analysis by Ren et al.

**Fig. 2.**
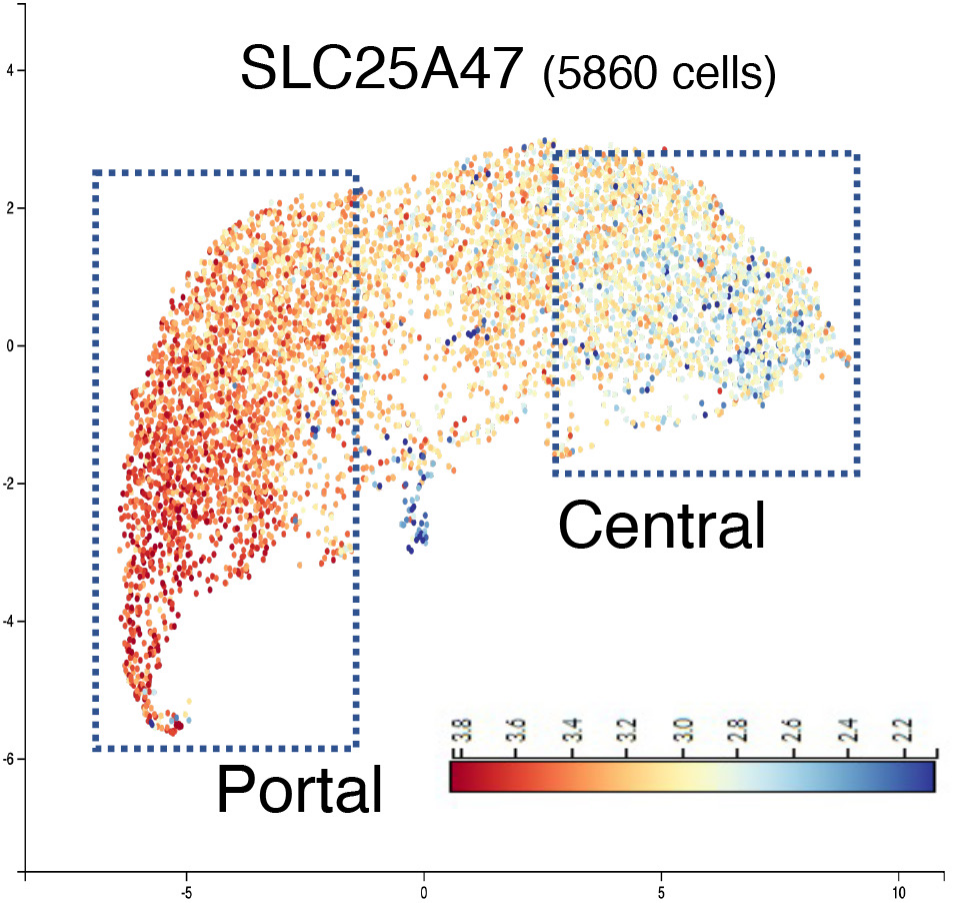
The spatial expression profile of *Slc25a47* in the mouse liver by Visium (from Liver Cell Atlas, https://www.livercellatlas.org). The portal and central regions are indicated. Horizontal and vertical axes indicate UMAP1 and UMAP2, respectively.

Third, cell-type matters. The liver is composed of heterogeneous cell populations, including hepatocytes, stellate cells, and immune cells, which all have variable transcriptional profiles (7). Notably, *Slc25a47* expression is highly enriched in hepatocytes around the portal vein, relative to a centrilobular distribution (Fig. 2). In contrast, the eQTL study by Ren et al. was based on bulk RNA-seq data and, therefore, would miss such cell-type-specific or location-dependent correlations. eQTLs with scRNA-seq might provide new insights into otherwise missing cell-type specific correlations (8, 9).

Fourth, the regulation of SLC25A47 is beyond transcription. One of the significant single nucleotide polymorphisms (SNPs) (rs35007880) is located in the coding region of the *SLC25A47* gene, which causes an Arg to Leu substitution (R135L) in the intermembrane domain of SLC25A47. This hydrophobic change may affect the transport activity, protein localization, or protein stability rather than the messenger RNA (mRNA) expression levels.

Lastly, we make no claims about whether SNPs in the *SLC25A47* locus are tracked with other SNPs, i.e., linkage disequilibrium. These SNPs may be more or less associated with certain populations; however, as discussed in a recent review (10), a strong dominant SNP effect within this region increases the likelihood of locus importance, irrespective of any eQTL findings. In fact, multiple highly significant SNP associations suggest that the *SLC25A47* locus is relevant to glycemic control in humans. Importantly, our functional studies in mice are consistent with the observation (4).

In conclusion, the significance of these SNPs on SLC25A47 function/expression should be evaluated by evidence-based wet-bench research.
